# Motorcycling can be dangerous: verrucous lichenoid reaction to a tattoo^☆^^☆☆^

**DOI:** 10.1016/j.abd.2020.07.013

**Published:** 2021-03-15

**Authors:** Isadora Rosan, Ninoska Nieto-Salazar, Marcello Menta Simonsen Nico

**Affiliations:** Department of Dermatology, Faculty of Medicine, Hospital das Clínicas, Universidade de São Paulo, São Paulo, SP, Brazil

**Keywords:** Allergic reaction, Lichenoid eruptions, Tattooing

## Abstract

Tattooing one’s body is currently a common practice worldwide; however, it is not risk-free. This is a case of a patient who tattooed himself motivated by his passion for motorcycles and then developed an exuberant lichenoid reaction to the red pigment used in the tattoo, with the appearance of verrucous lesions. Despite the lack of response to treatment, he states that he would tattoo his own skin again.

## Case report

Currently, the practice of tattooing is popular worldwide. This has led to an increase in reactions to tattoo pigments. These reactions are difficult to treat and can sometimes take on curious aspects.

**Figure 1:**
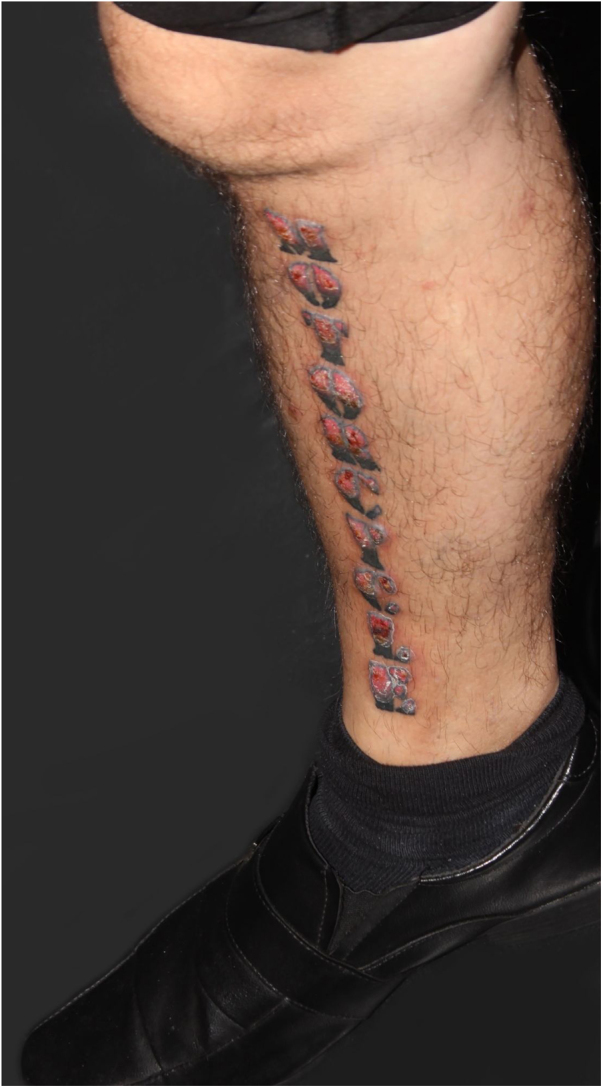
Clinical aspect of the tattoo approximately two months after the procedure: infiltration and inflammation of the red pigment area.

**Figure 2:**
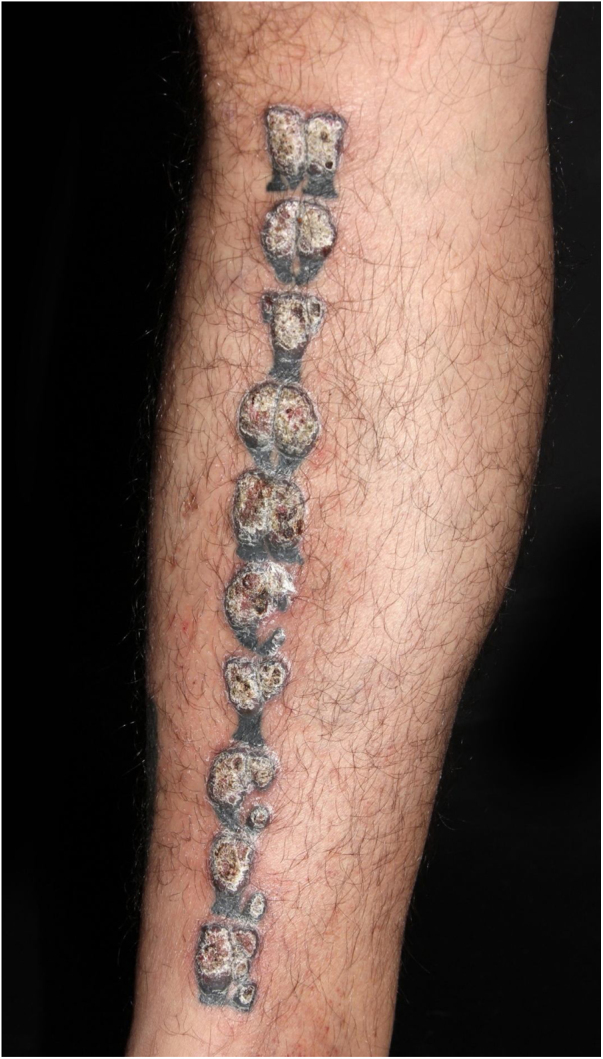
Clinical aspect after a few weeks: verrucous appearance of the regions tattooed with red pigment.

**Figure 3:**
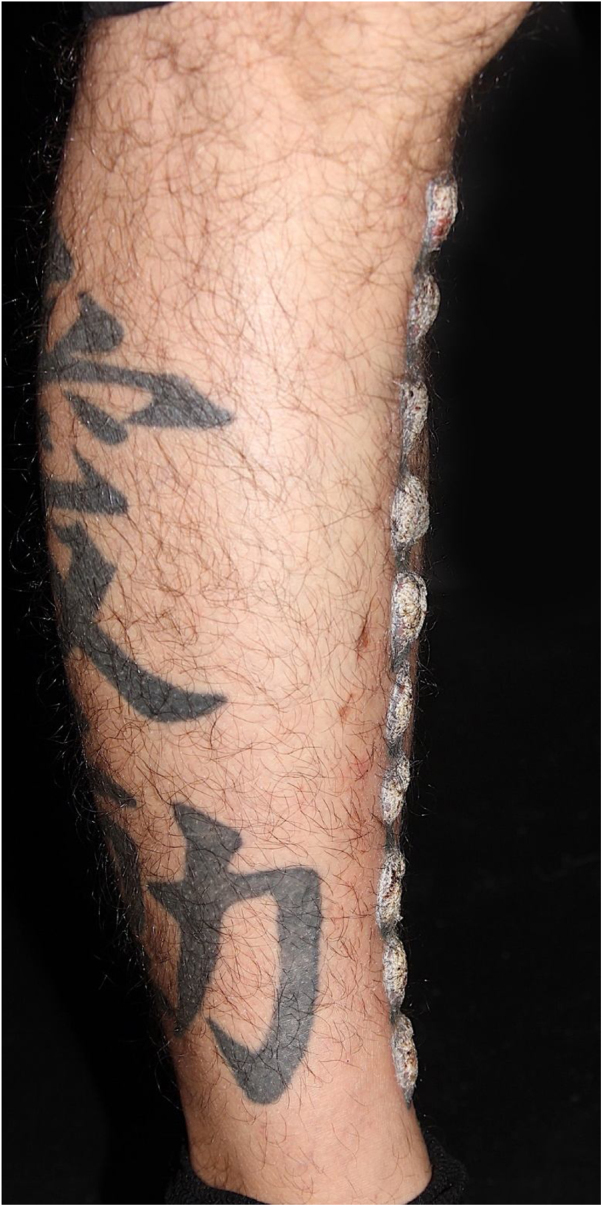
Lateral view of the verrucous lesions; notice a black tattoo with normal aspect on the lower leg.

**Figure 4:**
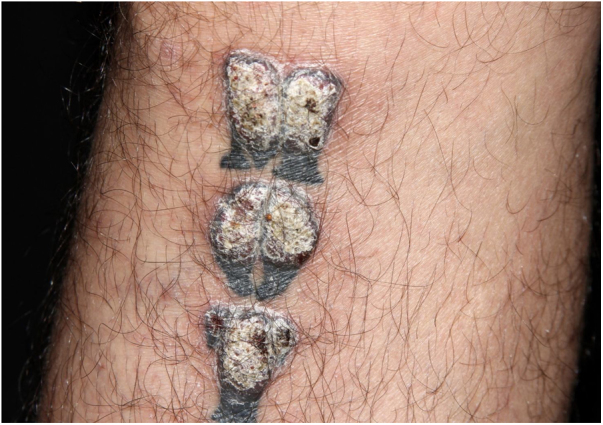
Closer view of the verrucous lesions - note that areas tattooed in black are spared.

A 39-year-old Brazilian male had his right leg tattooed with a 10-letter word representing his greatest passion: “motorcycle”. After 15 days, severe pruritus and inflammation appeared in the areas tattooed in red. The patient was assessed by our team approximately 2 months after the tattoo was performed; the dermatological examination showed infiltrated lesions in the areas tattooed in red and sparing the areas tattooed in black (Fig. 1). After a few weeks, these lesions took on an exuberant hypertrophic appearance, forming a bizarre “verrucous motorcycle” (Figure 2, Figure 3, Figure 4). Other areas tattooed in red located on the trunk and limbs also showed inflammation, but without the verrucous aspect seen on the leg. Other areas of these tattoos that were not red (blue, green, yellow, and black) were normal at the examination (Fig. 3). Several of these other tattoos also reflected the patient's love for motorcycles.

**Figure 5:**
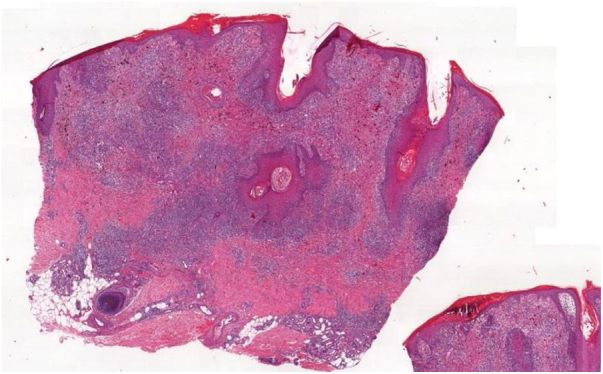
Histopathology of the verrucous area: acanthosis, superficial and deep inflammation, interface dermatitis and follicular aggression (Hematoxylin & eosin, ×40).

Histopathological analysis of the biopsy of one of the verrucous areas showed compact hyperparakeratosis, acanthosis, spongiosis, small lymphocyte exocytosis, and vacuolar degeneration of the basal layer. There was a nodular, diffuse and lichenoid infiltrate in the dermis, consisting predominantly of lymphocytes and histiocytes containing variable amounts of pigment. Marked inflammatory infiltrate aggression to the follicular epithelium was observed (Fig. 5). Special stains for microorganisms and tissue cultures for fungi and mycobacteria were negative. Immunohistochemistry showed: CD3 positivity in rare epidermal lymphocytes; CD4 and CD8 positivity in rare cells; CD4:CD8 ratio of 2:1; CD56 positivity in numerous mononuclear cells in the dermis. The final diagnosis of a lichenoid reaction to the tattoo red pigment was attained. Despite the lack of clinical response to the performed therapies (local infiltration of corticosteroids, and excision by shaving the verrucous lesions), the patient stated that he would certainly tattoo his skin again.

## Discussion

Even though tattoos constitute a disseminated practice, they are not entirely harmless and can result in both infectious and non-infectious complications.^1^ The pigment most often related to hypersensitivity reactions is the red one. The histopathological patterns that can be observed include eczematous, lichenoid, granulomatous, sarcoid, and pseudo-lymphomatous patterns.^2^ The composition of red pigments used in tattoos is varied and non-regulated. Recently, inorganic dyes in red tattoos have been replaced by organic ones, such as azo-compounds and quinacridone. Skin hypersensitivity reactions to organic red pigments can develop after a few weeks or even years.^3^ The patient's passion for motorcycles resulted in significant damage to his skin. Tattoos can be a creative way of expressing people's passions^3^; we, as dermatologists, must warn our patients about possible risks, even though our scientific explanations are often ineffective.

## Financial support

None declared.

## Authors’ contributions

Isadora Rosan: Data collection and writing of the manuscript.

Ninoska Nieto-Salazar: Data collection and writing of the manuscript.

Marcello Menta Simonsen Nico: Data collection and writing of the manuscript.

## Conflicts of interest

None declared.
